# Myelin characteristics of the corpus callosum in capuchin monkeys (*Sapajus* [*Cebus*] *apella*) across the lifespan

**DOI:** 10.1038/s41598-022-12893-z

**Published:** 2022-05-24

**Authors:** Chase M. Watson, Chet C. Sherwood, Kimberley A. Phillips

**Affiliations:** 1 Department of Psychology and Neuroscience Program, Trinity University, San Antonio, TX USA; 2 Department of Anthropology and Center for the Advanced Study of Human Paleobiology, The George Washington University, Washington, DC USA; 3 Southwest National Primate Research Center, Texas Biomedical Research Institute, San Antonio, TX USA

**Keywords:** Development of the nervous system, Glial development

## Abstract

The midsagittal area of the corpus callosum (CC) is frequently studied in relation to brain development, connectivity, and function. Here we quantify myelin characteristics from electron microscopy to understand more fully differential patterns of white matter development occurring within the CC. We subdivided midsagittal regions of the CC into: I—rostrum and genu, II—rostral body, III—anterior midbody, IV—posterior midbody, and V—isthmus and splenium. The sample represented capuchin monkeys ranging in age from 2 weeks to 35 years (*Sapajus* [*Cebus*] *apella*, *n* = 8). Measurements of myelin thickness, myelin fraction, and g-ratio were obtained in a systematic random fashion. We hypothesized there would be a period of rapid myelin growth within the CC in early development. Using a locally weighted regression analysis (LOESS), we found regional differences in myelin characteristics, with posterior regions showing more rapid increases in myelin thickness and sharper decreases in g-ratio in early development. The most anterior region showed the most sustained growth in myelin thickness. For all regions over the lifespan, myelin fraction increased, plateaued, and decreased. These results suggest differential patterns of nonlinear myelin growth occur early in development and well into adulthood in the CC of capuchin monkeys.

## Introduction

The corpus callosum (CC) is the major commissural white matter tract in the brain of placental mammals between the two cerebral hemispheres, establishing homotopic and heterotopic connections and playing a role in sensory, motor, and cognitive functions. The midsagittal area of the corpus callosum has frequently been studied in relation to brain development and maturation^1–4^. In humans, the CC undergoes tremendous growth during the first year of life with the total CC area and subdivisions of the genu, body, and splenium increasing by 40–100%^5,6^. Pujol et al.^3^ reported that the CC continues to increase in size until the mid-20s, although slower growth is seen after childhood. Danielsen et al.^7^, examined CC development in over 1000 participants ranging in age from 4 to 93 years and demonstrated accentuated growth through early adulthood. This growth was mainly driven by posterior regions of the CC including the splenium. Regional differences are apparent in these growth patterns, with the genu demonstrating a greater rate of increase during the first 6 years compared to the splenium, and the splenium demonstrating greater growth between 7 and 18 years^6^. A recent study reported rapid development of CC, showing quadratic association between age and myelin water fraction across most subdivisions^8^.

Nonhuman primates (NHPs) are critical comparative models for understanding lifespan changes of the brain and how responses to challenges during critical periods influence brain development with persistent effects on phenotype^9^. NHPs also show sustained increases in CC area during the first few years of life. In macaques (*Macaca mulatta*), total CC size increases from prenatal age to 32 weeks, with the splenium demonstrating the most growth^10^. Baboons (*Papio hamadryas*) also display continued growth of the CC until at least postnatal week 32, with the splenium showing the greatest growth and the genu and anterior midbody displaying the least maturation^11^. Multiple regions of the CC in capuchin monkeys (*Sapajus* [*Cebus*] *apella*) have been found to show an increase in midsagittal area from 4 days to 20 years (middle adulthood)^12^.

The morphology of the CC has also been examined across the lifespan, including later life development, in chimpanzees (*Pan troglodytes*) and baboons. In chimpanzees, a rapid increase during infancy is followed by a gradual increase in midsagittal CC area during the juvenile period and adolescence^13,14^. Westerhausen et al.^15^ also reported that the CC midsagittal area did not substantially decline from middle to late adulthood in chimpanzees. A similar pattern of slow, continuous growth and no decline in midsagittal area during late adulthood was reported for baboons^16^ (*Papio anubis*)*.*

The anatomical and functional relationship between microstructure and macrostructure of the corpus callosum is not fully understood. Aboitz et al., investigating the relationship between midsagittal area of the CC and fiber density, suggested a relationship between callosal area and the abundant, small fibers microstructure (in humans and rhesus monkeys). The microstructural changes within the CC that occur during development are poorly understood, though myelination processes are known to be involved. Myelination plays an important role in development, as it protects the axon from subsequent injury or damage and enables fast and efficient nerve conduction^17–19^. Miller et al.^20^ reported that the density of myelinated axons steadily increases in numerous cortical areas of chimpanzees and reaches adult-like completion by sexual maturity, whereas in humans, myelination was slower during childhood and continued beyond late adolescence. Geriatric common marmosets (*Callithrix jacchus*) display reduced myelin thickness and decreased number of myelinated axons in anterior regions of the CC compared to young adults^21^.

Here we investigated ultrastructural changes in myelin architecture within the CC of tufted capuchins from ages 2 weeks to 35 years old using electron microscopy. Tufted capuchins (*Sapajus* [*Cebus*] *apella*) are platyrrhine monkeys from South America noted for their complex manipulative abilities and foraging habits, making them an interesting comparative model for neurodevelopment^22,23^. In addition, previous studies have shown that capuchins display especially fast postnatal brain growth and rapid motor skill development. The neonatal capuchin brain is a smaller proportion of adult brain weight (c. 50%) than is the brain of any other primate except humans and great apes, indicating that a large fraction of volumetric growth occurs in the early phase of postnatal life^24^. We used post-mortem tissue in a cross-sectional sample population to explore age-related changes in axons and myelin. We hypothesized capuchins would demonstrate dynamic changes in myelin characteristics across the lifespan, with a rapid increase in myelin during early development among all subdivisions of the CC.

## Methods

### Specimens

We used formalin-fixed brain samples of eight capuchin monkeys (*Sapajus *[*Cebus*] *apella*) ranging in age from 2 weeks to 35 years (see Table 1). All subjects were socially housed and only involved in behavioral and cognitive research studies at either the Southwest National Primate Research Center (Texas Biomedical Research Institute, San Antonio, Texas) or Hiram College (Hiram, Ohio). All research procedures were approved by the Institutional Animal Care and Use Committee at Southwest National Primate Research Center or Hiram College. All methods reported are in accordance with ARRIVE guidelines. All animals were maintained in accordance with Federally recognized standards, guidelines, and principles. Cause of death included such conditions as acute respiratory illness, mortal injury sustained after an aggressive social interaction, or euthanasia as a result of failing health due to old age. As none of the causes of death were neurological in nature, we do not believe the measures of myelin were affected. Between 1 and 6 h after death, the brain was removed and immersed in 10% formalin. After an initial fixation period of 7–10 days, brains were transferred into 0.1 M phosphate buffered saline (PBS) with 0.1% sodium azide solution and stored at 4 °C until processing for electron microscopy.

**Table 1:** Characteristics of the capuchin monkey brains used in the study.

Animal ID	Age (in years)	Sex
M1	0.04	M
M2	0.55	M
M3	0.55	M
F1	5.57	F
M4	6.71	M
M5	9.90	M
M6	16.66	M
F2	35.45	F

### Electron microscopy

**Figure 1:**
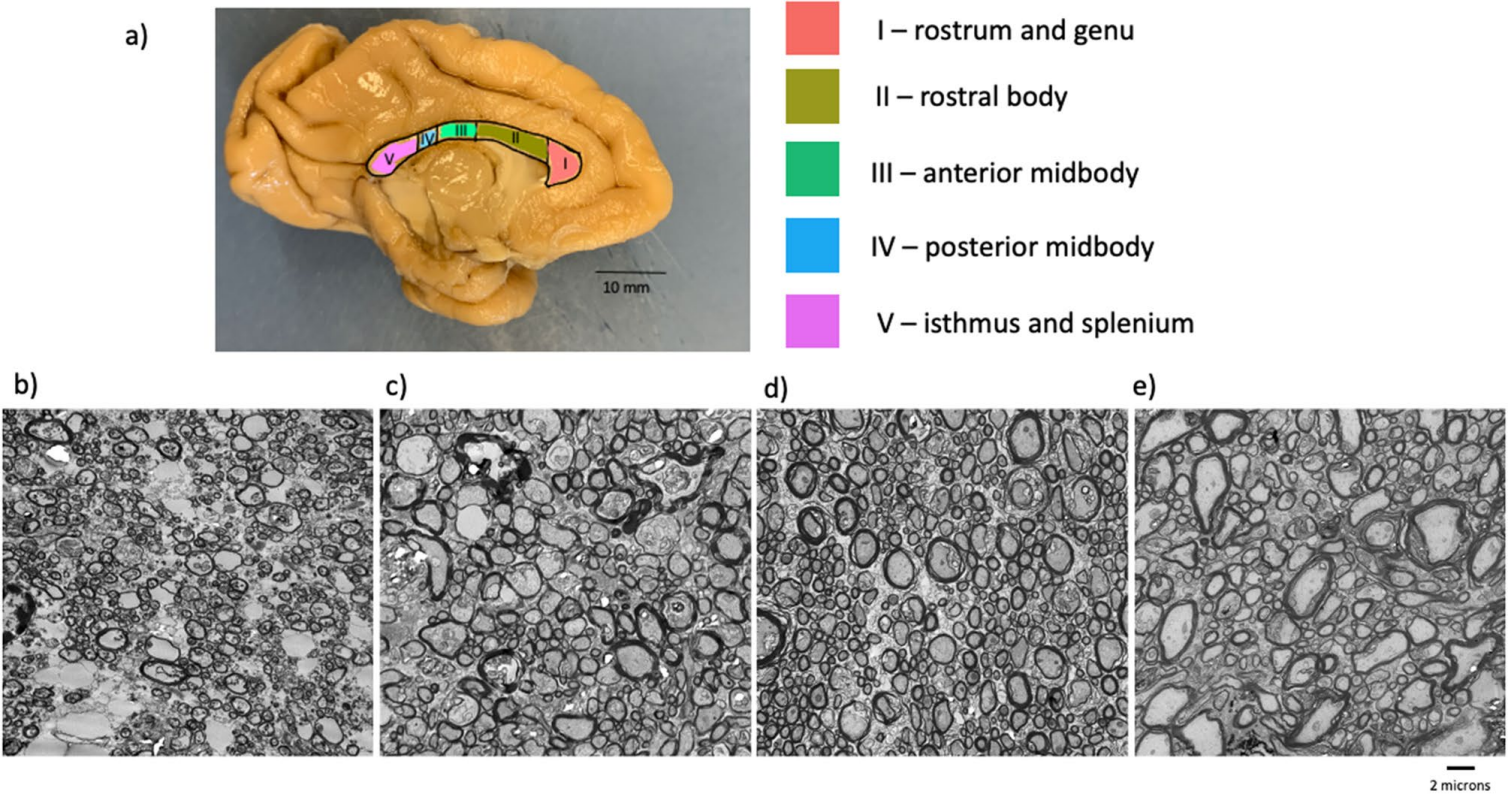
(**a**) The corpus callosum of a capuchin (*Sapajus* [*Cebus*] *apella*), indicating the five regional subdivisions (I–V). Electron micrographs of myelinated axons in Region V of subjects at (**b**) 0.55 years, (**c**) 5.60 years, (**d**) 9.90 years, and (**e**) 35.40 years.

All brains were bisected at the midsagittal plane and the CC was sectioned from the left hemisphere of each specimen. The CC was then subdivided into five regions, guided by prior studies which used DTI tractography to identity functional divisions of the CC in nonhuman primates^25,26^. The resulting classification follows Hofer and Frahm’s scheme^25^; these regions comprise the following fiber projections: I = prefrontal lobe (most anterior segment covering 1/6 of the CC), II = premotor and supplementary motor cortices (the remaining portion of the anterior half), III = motor cortex (the posterior half minus the posterior 1/3), IV = sensory cortex (posterior 1/3d minus the posterior ¼), V = parietal, temporal, and occipital lobes (posterior ¼ of the CC; see Fig. 1a).

A 1 mm × 2 mm × 1 mm piece was cut from the center of each region and placed into a microcentrifuge tube containing 1% glutaraldehyde and 4% paraformadehyde, for a period of at least 1 week. Samples were prepared for electron microscopy using a modified technique for processing nerve biopsies^9,21^. Samples were postfixed in 1% Zetterqvist’s buffered osmium tetroxide, dehydrated, infiltrated with resin and embedded before sectioning. Semithin sections were then cut and stained with toluidine blue for examination under a light microscope to verify orientation before continuing. Subsequent ultrathin sections were mounted on electron microscopy grids and stained with uranyl acetate before viewing under the electron microscope.

Myelin characteristics were determined from electron micrographs from 5000 × (with the exception of region I from Subject X and region III from Subject x, which were 10,000 ×) digital images taken from a digital camera coupled to a JOEL JEM 1230 electron microscope. An average of 353 axons (± 59) were measured from each CC subdivision for each subject. Counts and measurements of axon diameter were made over 26.3 × 26.3 µm^2^ and 13.2 × 13.2 µm^2^ regions in a systematic‐random fashion using fractionator sampling implemented in ImageJ software (ImageJ version 1.48). An average of 26 (± 3) sampling sites were visited per region of interest.

Axon diameter was defined as the average of the fitted major and minor axis lengths for major‐minor ratios, and otherwise as the minor axis^27^. G-ratio is defined as the ratio between the inner axon diameter and the outer myelinated axon diameter^28^ and provides an estimate of how much of the axon is myelin in proportion to its diameter. A smaller g-ratio indicates a larger proportion of myelin.

Myelin fraction (MF) quantifies the proportion of area consisting of myelin to non-myelin in a sampling frame. To estimate MF we used a method of automated image thresholding and binarization similar to what others have used to quantify neuropil fraction, and allows one to quantify the proportion of an area consisting of myelin^29,30^ and as developed and utilized in previous work^21^. Measurements were conducted by CMW, who was blind to age and sex of subjects; validation checks were conducted by KAP.

### Statistical analyses

As our sample size was quite small, we were unable to perform statistical analyses to quantify regional differences in growth patterns of the CC. To examine developmental growth patterns of myelin thickness, myelin fraction, and g-ratio, we used the nonparametric local smoothing model called locally weighted regression analysis (LOESS)^31^. Unlike parametric regression models, this method fits the data locally for each point, x, using a neighborhood of points (the size of which is controlled by a smoothing parameter, α), weighted by their distance from point x. Therefore, LOESS does not make any assumptions about the structure of the data, and is ideal for modeling growth curves with small sample sizes. This method has been commonly used to model growth in comparative biology studies of primates^32–34^. Using this model allowed us to apply curve fitting to relatively few data points without using a parametric function. A fitting procedure was used to avoid distortion of the smoothing function by deviant data points^35^. Data were fitted using alpha = 1. LOESS fit depends upon the smoothing parameter used. A smaller smoothing parameter will result in overfitting and the fit will interpolate the data. If the smoothing parameter is too large, the fit will be excessively smoothed. Ideally, the smoothing parameter selected will be between these two extremes. The strategy we used to set the smoothing parameter at 1 was to examine plots of the fit residuals associated with the predictor variable; we then chose the largest smoothing parameter (1) that yielded no clearly discernible information in the fit residuals.

For all LOESS analyses, the oldest subject (35 years) was excluded. As the next oldest subject was 16 years, we considered the 35-year-old to be an outlier due to the large age gap. The data from the 35-year-old are visualized on the Figures. RStudio Version 1.2.5001 was used for all analyses and data visualization^36^.

## Results

Electron micrographs of representative tissue from young and adult capuchins are presented in Fig. 1b–e. The young juvenile capuchins (Fig. 1b) had only a few, thinly myelinated axons. Young adult capuchins (Fig. 1c,d) had thicker myelination and a greater density of myelinated axons. Degradation of myelin, indicated by layers splitting, can be seen in some axons in the oldest subject (Fig. 1e).

### Myelin thickness

**Figure 2:**
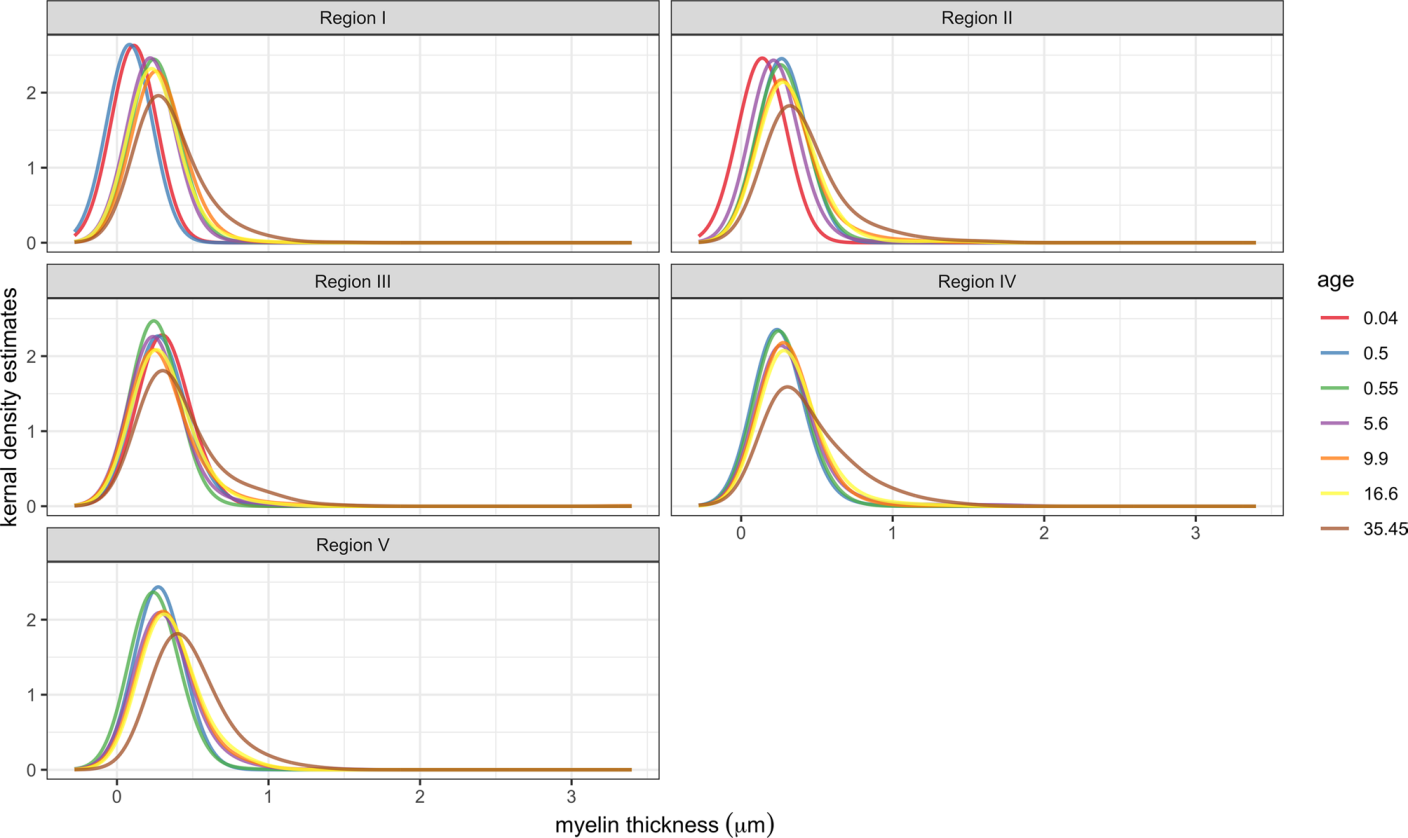
Kernel density estimation plot of myelin thickness measurements in callosal Regions I–V. Subjects are referred to by age in years. Youngest subject (0.04 years) was excluded from Regions IV and V, as no myelinated axons were found in these regions.

**Figure 3:**
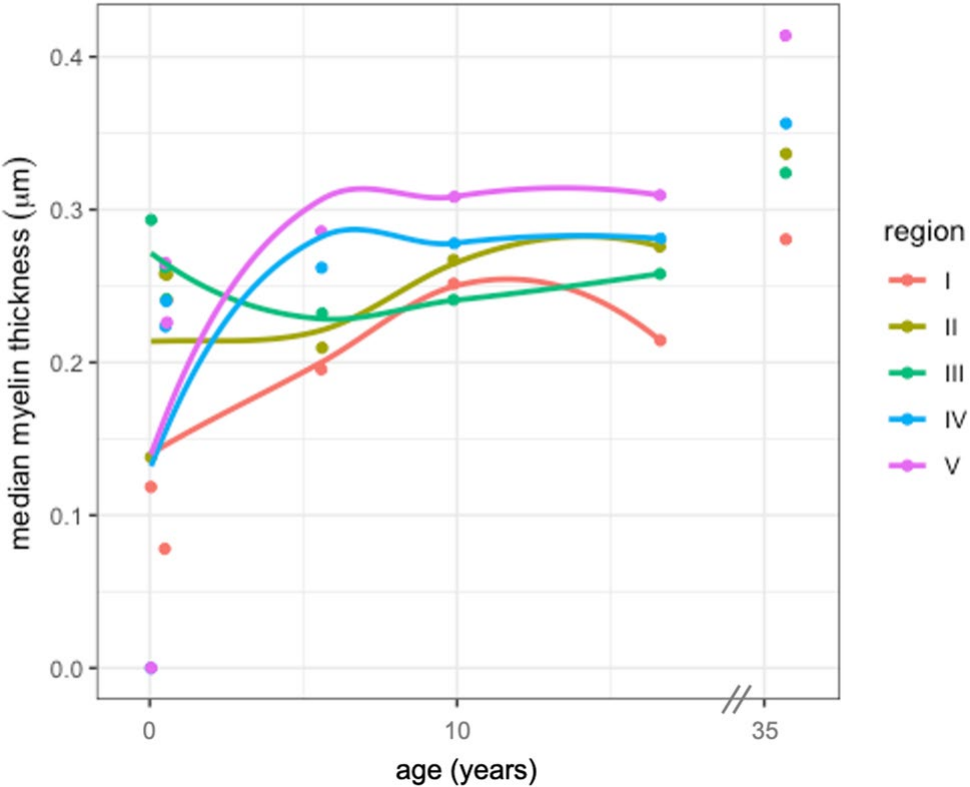
Scatterplot of median myelin thickness by age seperated by region. The lines were fit with LOESS regression. One subject (35.4 years) was excluded from LOESS regression.

Across all regions, the most frequently occurring myelin thickness was 0.2 µm; myelin thickness greater than 0.4 µm was observed infrequently. While there was variability in the range of myelin thickness across ages and regions, the distribution displayed a positive skew (Fig. 2). Myelin thickness showed variable patterns of nonlinear growth depending on region (see Fig. 3), with posterior regions (Regions IV and V) displaying the most rapid increase in early development. Region I showed a sustained increase in myelin thickness through early adulthood. Myelination in Region II displayed a later onset of increased growth. Region III showed a more stable pattern of myelin thickness across development.

### G-ratio

**Figure 4:**
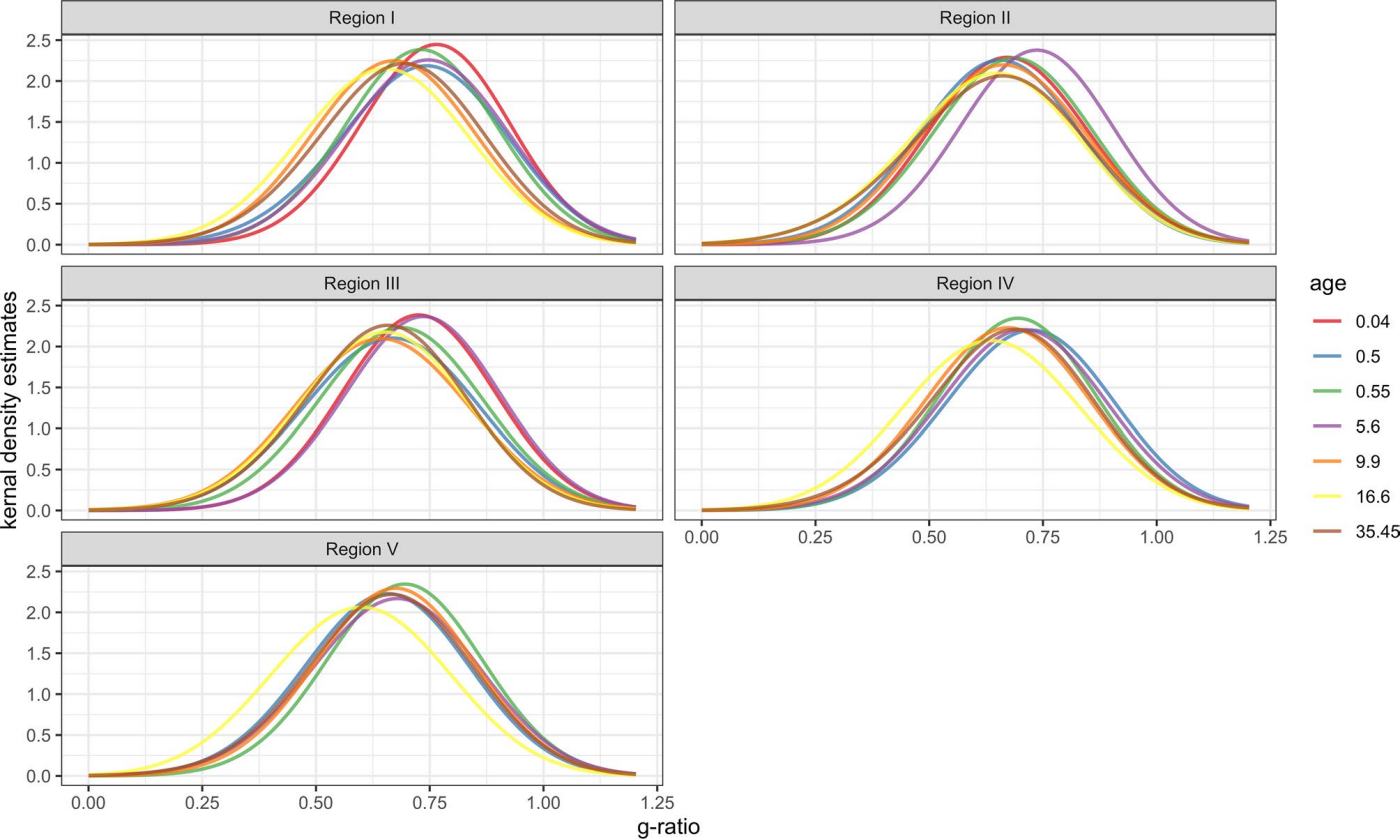
Kernel density estimation plot of g-ratio measurements in callosal Regions I–V. Subjects are referred to by age in years. The youngest subject (0.04 years) was excluded from Regions IV and V, as no myelinated axons were found in these regions.

**Figure 5:**
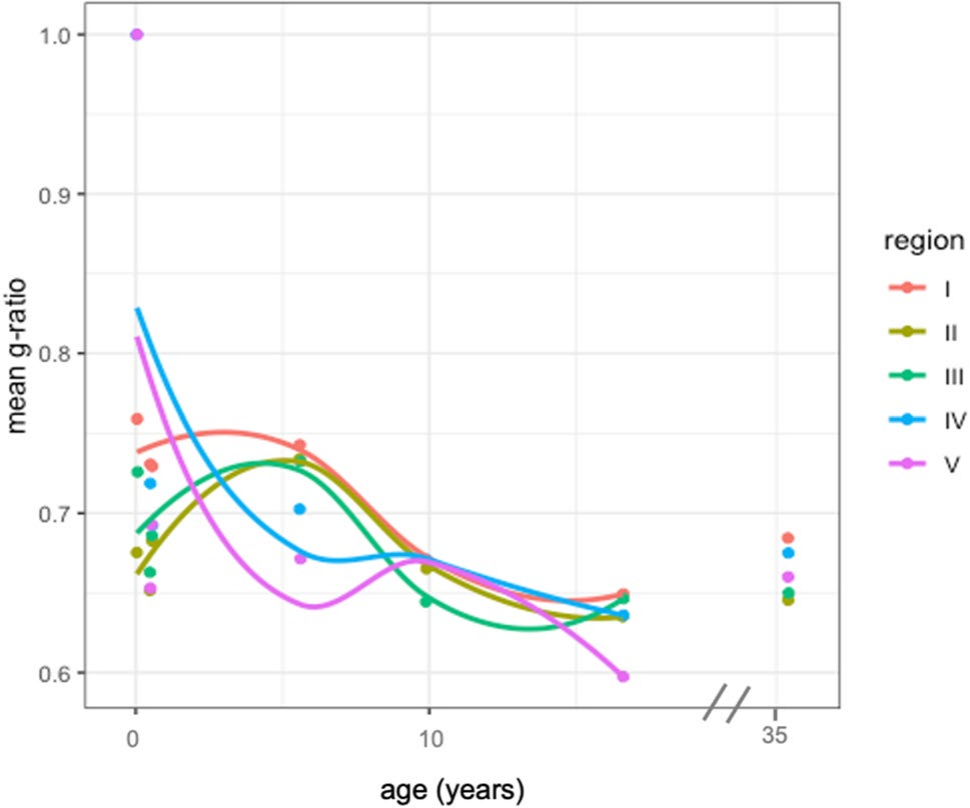
Scatterplot of mean g-ratio by age seperated by region. The lines were fit with LOESS regression. One subject (35.4 years) was excluded from LOESS regression.

The distribution of g-ratio across ages and regions was approximately normal and varied little by region of the corpus callosum (Fig. 4). LOESS fits of scatter plots for the mean g-ratio by region indicate that for most regions, the g-ratio followed a stable trajectory over the lifespan and then slightly decreased with subject age (Fig. 5). However, regions IV and V showed a sharper decrease in g-ratio in early development, indicating an increase in myelin in proportion to axon diameter.

### Myelin fraction

**Figure 6:**
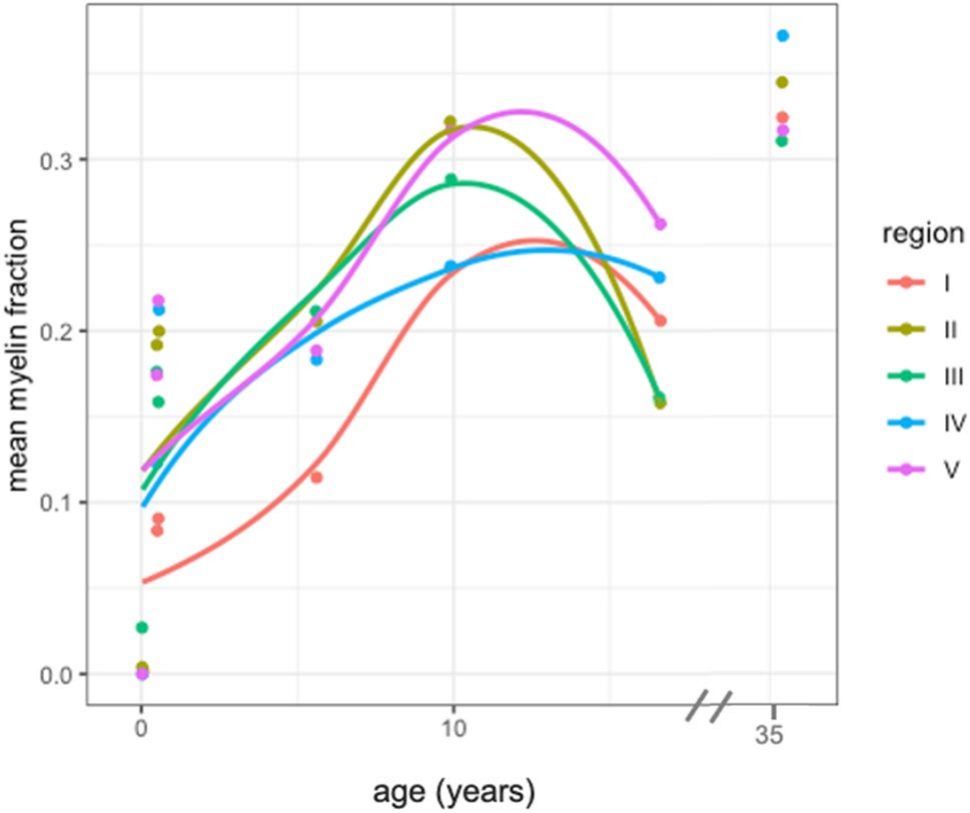
Scatterplot of mean myelin fraction by age seperated by region. The lines were fit with LOESS regression. One subject (35.4 years old) was excluded from LOESS regression.

LOESS fits of scatterplots of the developmental patterns of MF indicate all regions displayed a similar rapid increase in MF, followed by a plateau, and then a small decrease (see Fig. 6).

## Discussion

The early postnatal period in capuchins is a dynamic period of white matter development, with changes in myelin characteristics especially pronounced in anterior and posterior regions of the CC. Our data also illustrate the pattern of age-related differences in myelin characteristics (myelin thickness, g-ratio, and myelin fraction) across the lifespan of capuchins. As capuchins attain sexual or reproductive maturity between 4 and 10 years^37,38^, these data suggest that white matter growth in the CC continues past adolescence and into early adulthood. Capuchins share this pattern of continued white matter development with humans and chimpanzees^39–41^. However, humans appear to have the greatest prolonged myelination^20^ and show atrophy of the CC in later life^7,15^.

Studies in humans have reported greater age-related changes in the midsagittal CC area of posterior regions than in anterior regions during late childhood and early adolescence^42–44^. However, when exploring early childhood development, it appears anterior growth may be more prominent^44^. Taken together, it has been hypothesized that there is likely an anterior-to-posterior maturation gradient in humans, with posterior maturation taking place later than anterior growth^45^. Our data indicate capuchins display a different regional pattern of myelin development, with posterior regions of the CC showing relatively rapid growth of myelin characteristics during early life, reaching a plateau around age 6 years (adolescence) and anterior regions showing slower but sustained growth.

These patterns of myelin development demonstrate the dynamic process of white matter growth, which likely involves the fine-tuning of axonal connections among cortical areas across the cerebral hemispheres. Other findings have also observed sustained growth patterns in both anterior and posterior regions in the CC of capuchins^12^. The splenium, which is most posterior, is important for visuospatial processes, as it connects occipital and temporal cortices. Furthermore, at least in humans, auditory and possibly attentional resources are also mediated via the splenium^46,47^. The genu, the most anterior region, is important for higher order cognitive functioning. In the wild, developing capuchins likely rely heavily on the connections of these regions for their complex foraging behaviors, involving prey capturing^48^, skilled tool use^49^, and the creation of complex mental maps of food sources^50^.

Effects of myelin growth on neuronal signaling processes can be interpreted in the context of the effects of changes in the relationship between axon and myelin dimensions. The g-ratio, which is the ratio of the inner to the outer radius of a myelinated axon, has been shown to affect signal conduction velocity^51^. The g-ratio values in our study sample followed a fairly normal distribution, and our values were consistent with the reported g-ratio values for other non-human primates such as macaques^52^. Our data suggest that average g-ratio decreases across the lifespan in the CC of capuchins, particularly in posterior regions, but the overall g-ratio range remains fairly constant with age. Similarly, some findings report that the g-ratio also decreases across the lifespan in humans. In humans, a decrease in g-ratio has been reported to occur during early development^53^ while another study reported that g-ratio remains constant with age^54^. Thus, while myelin thickness and myelin fraction appear to increase in some regions, these effects on conduction velocity may be somewhat counterbalanced by changes in axon diameter to maintain an optimal g-ratio distribution. Future work is needed to examine how changes in axon myelination and diameter across the lifespan coordinate the speed and efficiency of neuronal signaling processes.

While our findings offer novel insight on ultrastructural changes of the CC in capuchins, there are limitations. First, our small sample size created inconsistent gaps in the age distribution. Thus, our data were unlikely to detect small differences with age and were likely less able to balance the effects of individual variation. It should also be noted that the sample consisted predominantly of males. Corpus callosum morphology in capuchins has been reported to be influenced by sex^55^, with adult females having a larger overall CC than males. Nevertheless, our study is valuable as it is rare to obtain neurodevelopmental ultrastructure data in nonhuman primates. As data that are more representative of age and sex become available, a clearer picture will emerge on whether there are sex differences in the development of capuchin myelin characteristics.

To our knowledge, our study is the first to characterize developmental patterns of myelin in the CC of capuchins. While most other studies have explored morphometric changes in the CC from MRI scans, our method allowed for a detailed examination of fiber composition and myelination processes. The results of our examinations suggest that a prolonged nonlinear development of myelination occurs in the CC of capuchins, with regional variation in growth trajectories. However, the effects of these changes on the processing speed of these axonal connections and the subsequent effects on behavior should be further explored. These changes in myelin characteristics could be implicated in the complex behaviors that capuchins display more frequently as they mature. These findings also have relevance for understanding the evolutionary context of CC development in humans. Primates such as chimpanzees, macaques, and capuchins, all share several important characteristics of development with humans, including a prolonged infancy and juvenile period, complex manipulative abilities and foraging habits, and complex social behavior^56–58^. Therefore, nonhuman primates such as capuchins could serve as good translational models to better understand the developmental trajectory of the human brain.

## Data Availability

The authors confirm that the data supporting these findings and figures are available within this article and are available from the corresponding author, upon reasonable request.
